# Double-Plate Technique for Long-Construct Anterior Cervical Discectomy and Fusion

**DOI:** 10.7759/cureus.47407

**Published:** 2023-10-20

**Authors:** Jacob Razzouk, Debra Cheng, Ethan Vyhmeister, Olumide Danisa, Wayne Cheng

**Affiliations:** 1 Department of Orthopaedic Surgery, Loma Linda University Medical Center, Loma Linda, USA; 2 Division of Orthopaedic Surgery, Jerry L. Pettis VA Medical Center, Loma Linda, USA

**Keywords:** plate, cervical spine, plating technique, anterior cervical discectomy and fusion (acdf), double plate

## Abstract

The standard technique for multilevel anterior cervical discectomy and fusion (ACDF) uses a single plate to span multiple vertebral levels. However, the usage of single long plates is linked to potential hardware failure and screw pullout from stress overload. A single long plate is also more likely to fail at the caudal levels. Furthermore, centering a long plate spanning multiple levels requires simultaneous exposure to anatomy that may require more traction, technical expertise, and a potential increase in operative time. The use of a double-plate technique may be less technically demanding and, at the same time, allow for future revision to be confined to a shorter segment rather than requiring the removal of the entire single plate. In this study, we describe a surgical technique that involves using two plates during three or more levels of ACDF, discussing its advantages and limitations.

## Introduction

Anterior cervical discectomy and fusion (ACDF) is one of the most frequently performed spinal procedures in the United States, with approximately 137,000 ACDFs performed annually [1-3]. The standard technique for multilevel ACDF uses a single plate to span multiple vertebral levels [1,4,5]. However, the usage of single long plates is linked to potential hardware failure and screw pullout from stress overload [6]. A single long plate is also more likely to fail at the caudal levels, resulting in kyphosis or nonunion [7]. Furthermore, centering a long plate spanning multiple levels requires simultaneous exposure to anatomy that may require more traction, technical expertise, and a potential increase in operative time. The use of a double-plate technique may be less technically demanding and, at the same time, allow for future revision to be confined to a shorter segment rather than requiring the removal of the entire single plate. In this study, we describe a surgical technique utilizing two plates during three or more levels of ACDF, discussing its advantages and limitations.

## Case presentation

The double-plate technique for a long anterior cervical construct is described as follows. 

Patient positioning and exposure

The patient is placed in the supine position with 10 pounds of halter traction. A traditional extensile approach anterior to the sternocleidomastoid muscle may be used. Alternatively, two separate horizontal incisions following Langer’s lines can be used for cosmetic advantages.

Plate placement

Select the shortest plate to span the pathology. Usually, for a three-level construct such as C3 to C6 ACDF, a one-level plate is used for C3-C4 and a two-level plate is used for C4-C6. For a four-level construct such as C3-C7, a two-level plate for C3-C5 and a second two-level plate between C5 and C7 may be used. 

Case 1

**Figure 1:**
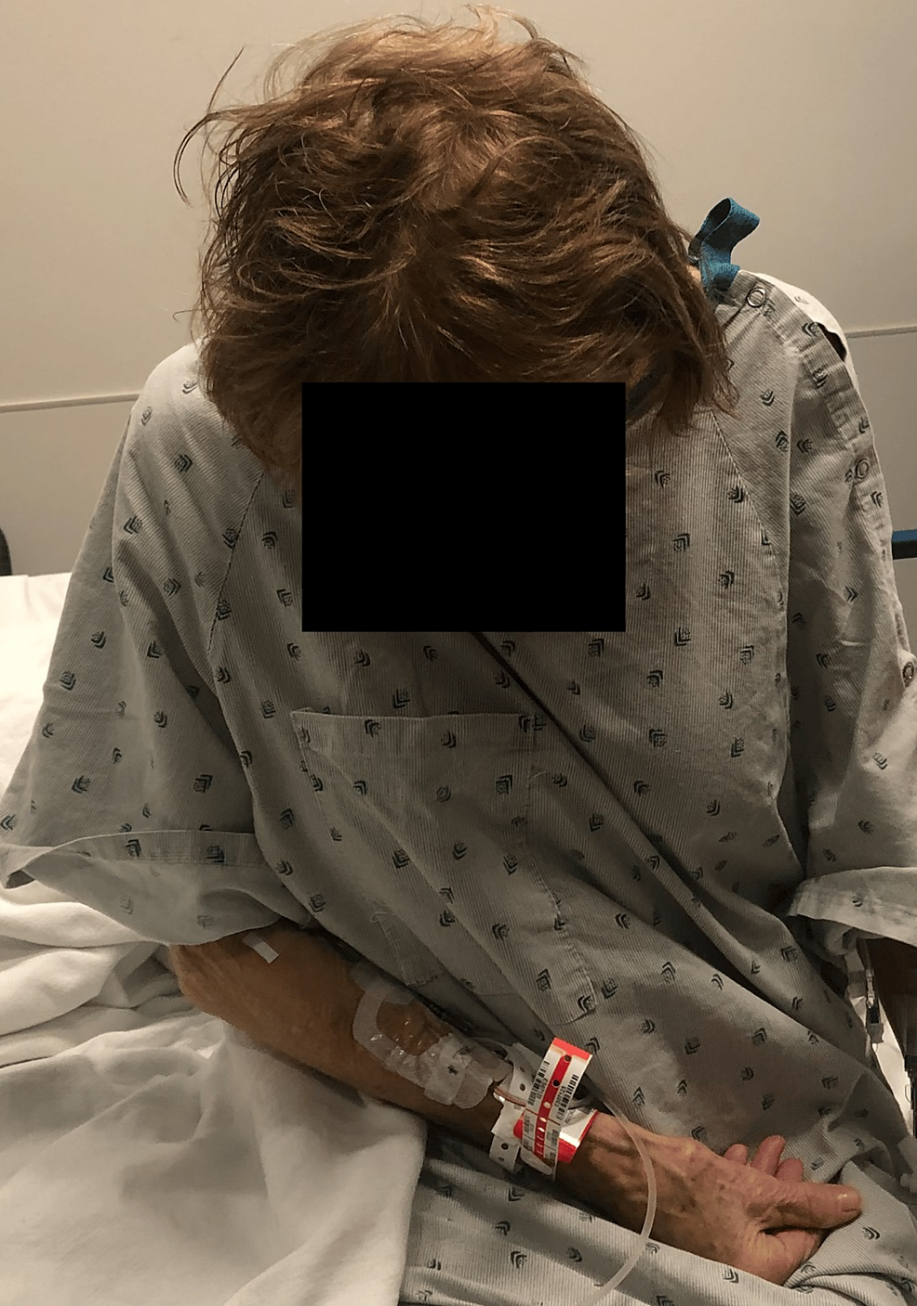
AP preoperative appearance of case 1. This is an AP photograph of the patient demonstrating a chin-on-chest deformity. AP, anterior-posterior

**Figure 2:**
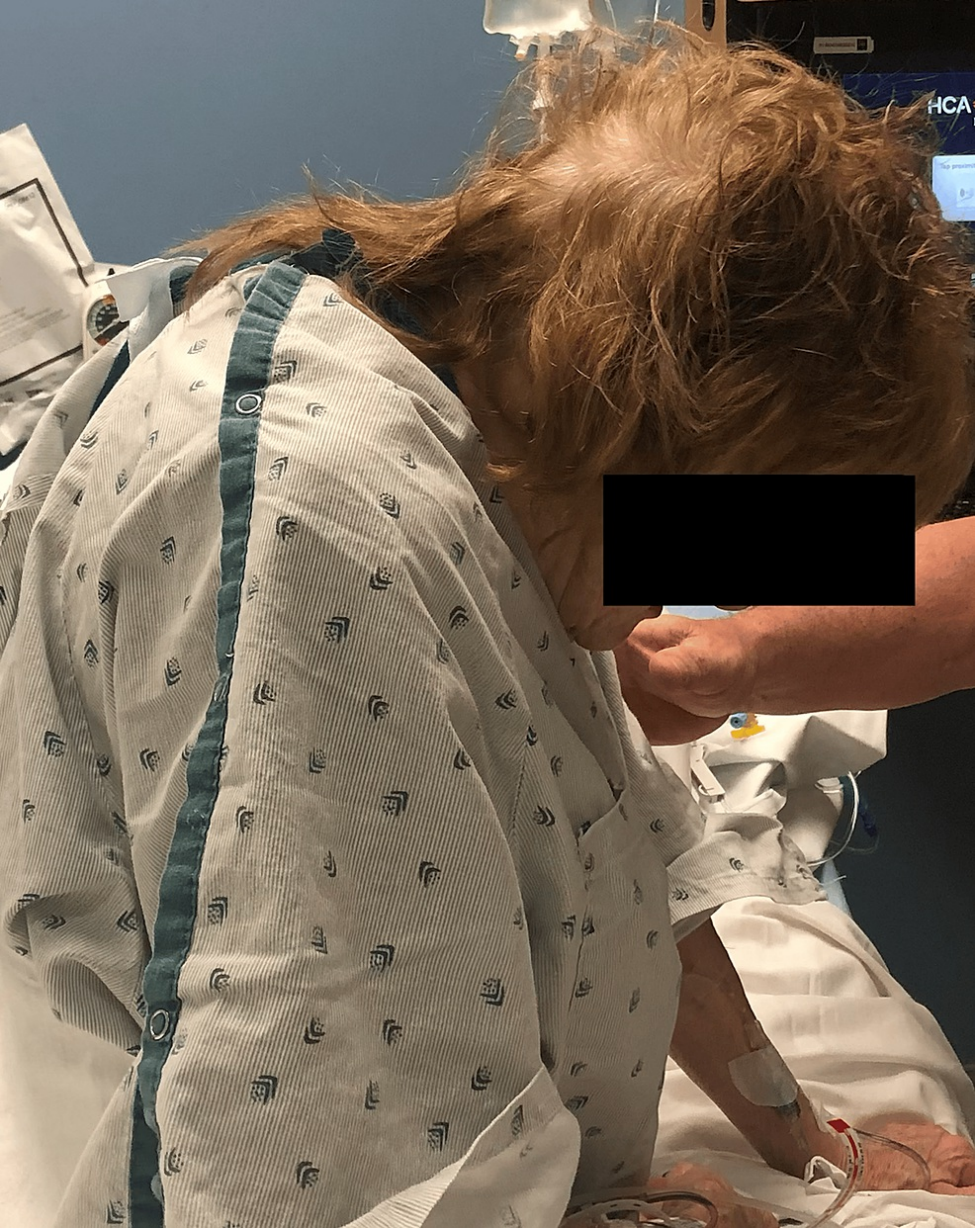
Lateral preoperative appearance of case 1. This is a lateral sagittal profile demonstrating the preoperative chin-on-chest deformity.

**Figure 3:**
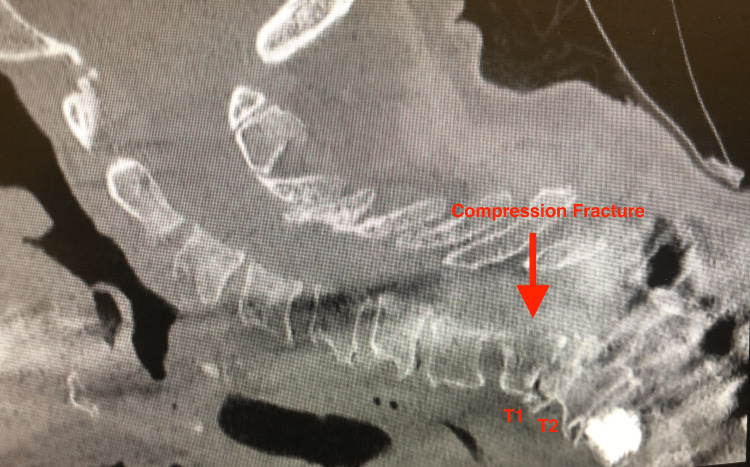
Illustration of a compression fracture in case 1. This is a sagittal cervical spine CT, with the arrow denoting a T1 compression fracture with spondylolisthesis of T1 on T2. CT, computed tomography

**Figure 4:**
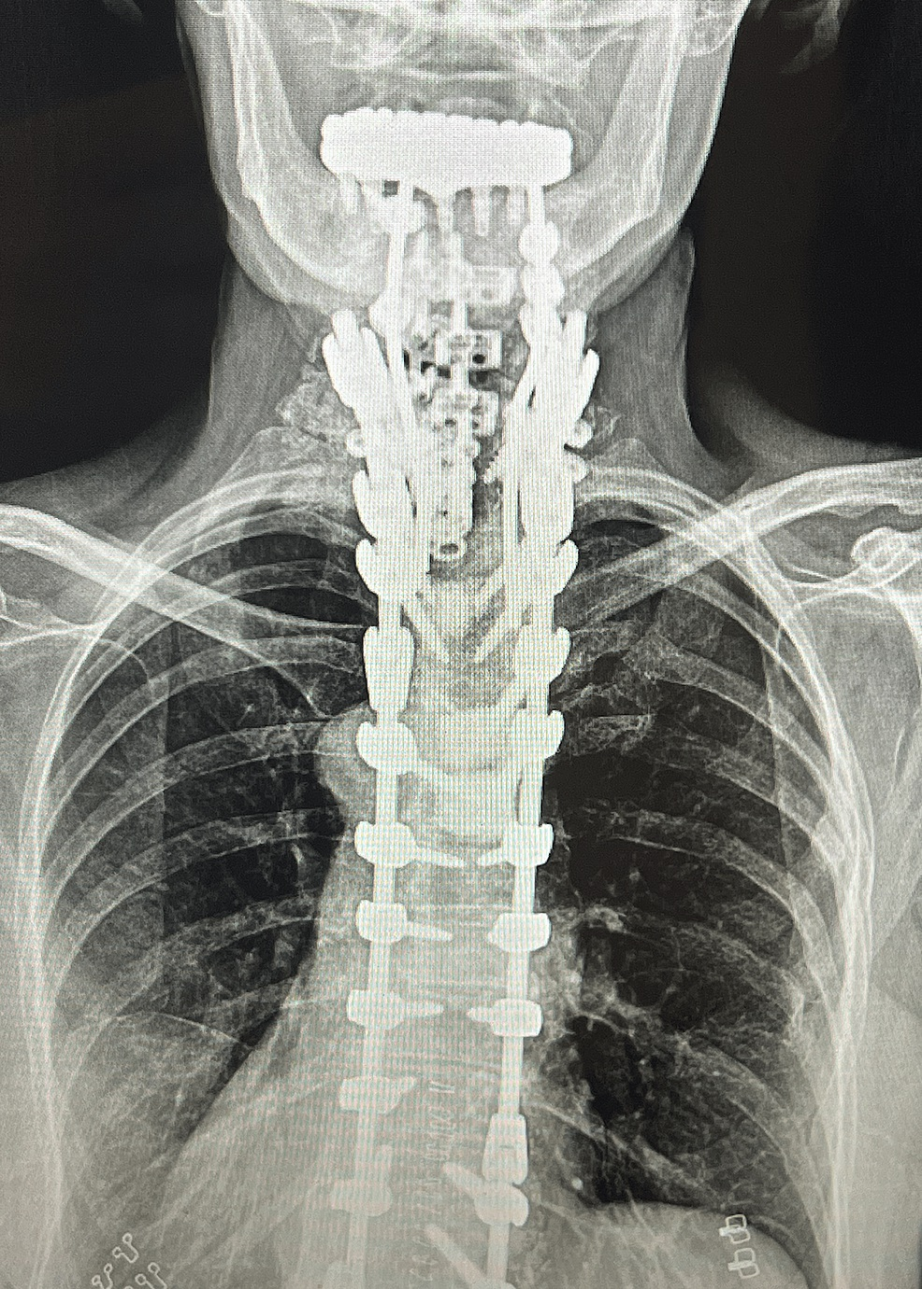
AP postoperative plain film imaging. This is an AP radiograph of the cervical and thoracic spine following the double-plate technique. AP, anterior-posterior

**Figure 5:**
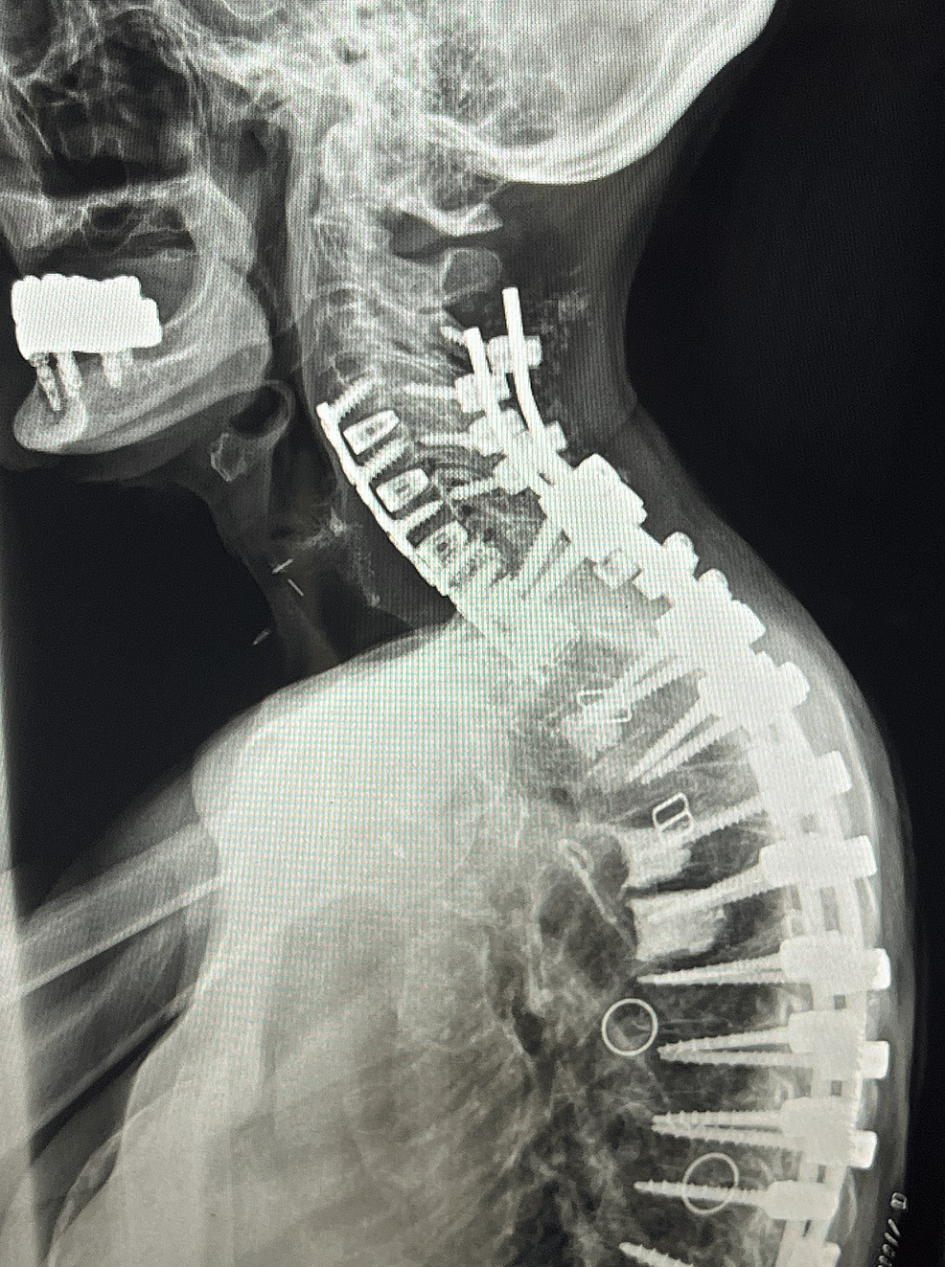
Lateral postoperative plain film imaging. This is a lateral radiograph of the cervical and thoracic spine demonstrating the postoperative double-plate technique.

The first patient was a 66-year-old female with a body mass index (BMI) of 18.21 kg/m^2^ (1.67 m, 50.8 kg) and a chief complaint of severe neck pain (pain scale 9/10) and inability to extend her neck. The preoperative appearance of case 1 is illustrated in anterior-posterior (AP) and lateral views in Figures 1-2, respectively. The patient had kyphoscoliosis and underwent T2-to-pelvis reconstruction. Postoperatively, a compression fracture with kyphotic deformity at T1 above the most cephalad instrumented level occurred. Figure 3 illustrates the compression fracture. The patient underwent a T1 corpectomy, C4-T2 anterior fusion, and instrumentation with the double-plate technique. The cephalad plate spanned from C4 to C7, and the second plate spanned from C7 to T2. Figures 4-5 display her postoperative AP and lateral imaging. The patient reported an improvement in visual analog scale (VAS) from 90 preoperatively to 30 postoperatively at one-year follow-up.

Case 2

**Figure 6:**
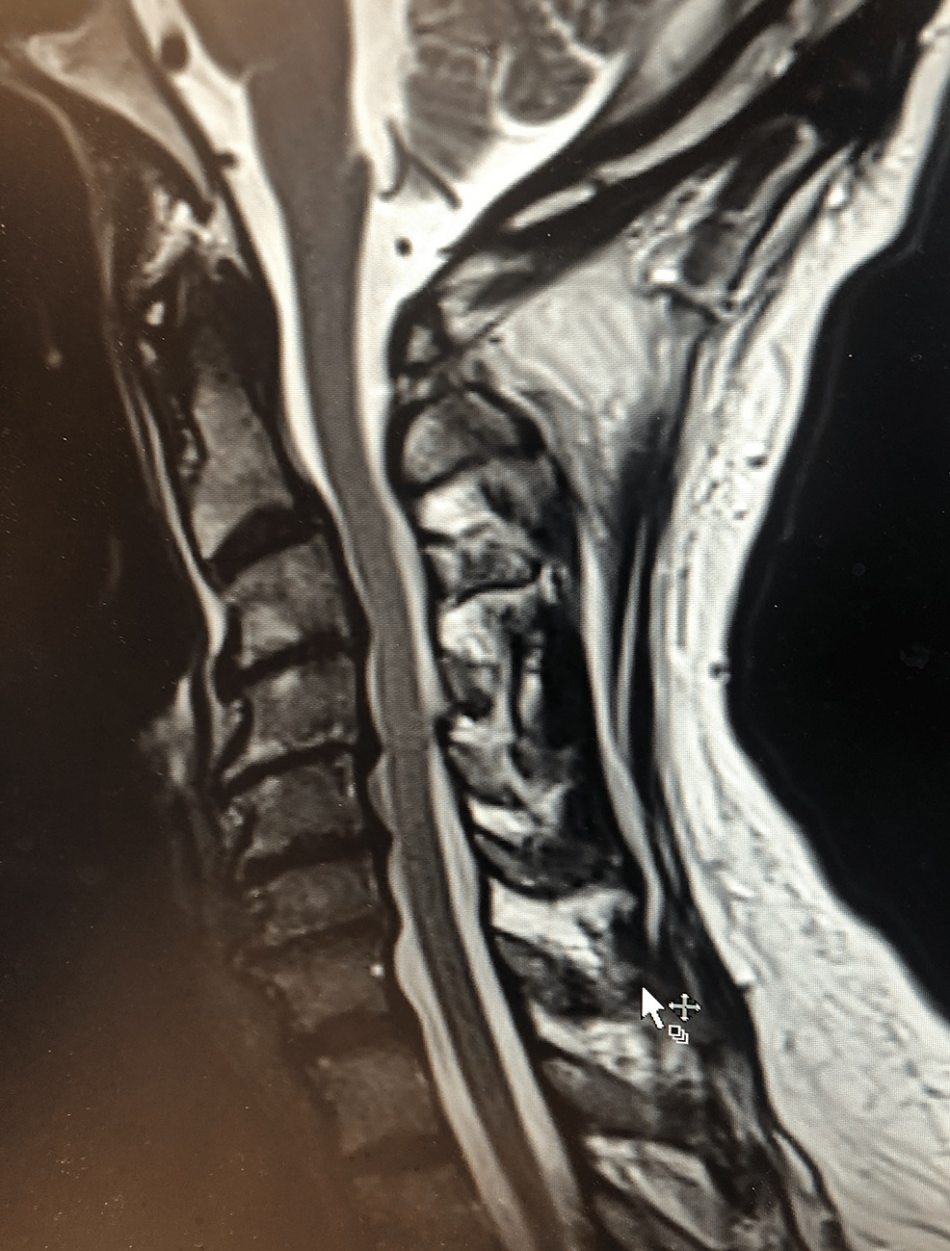
Preoperative sagittal T2-weighted MRI. This is a preoperative MRI of the cervical spine demonstrating spondylosis between C3 and C7. MRI, magnetic resonance imaging

**Figure 7:**
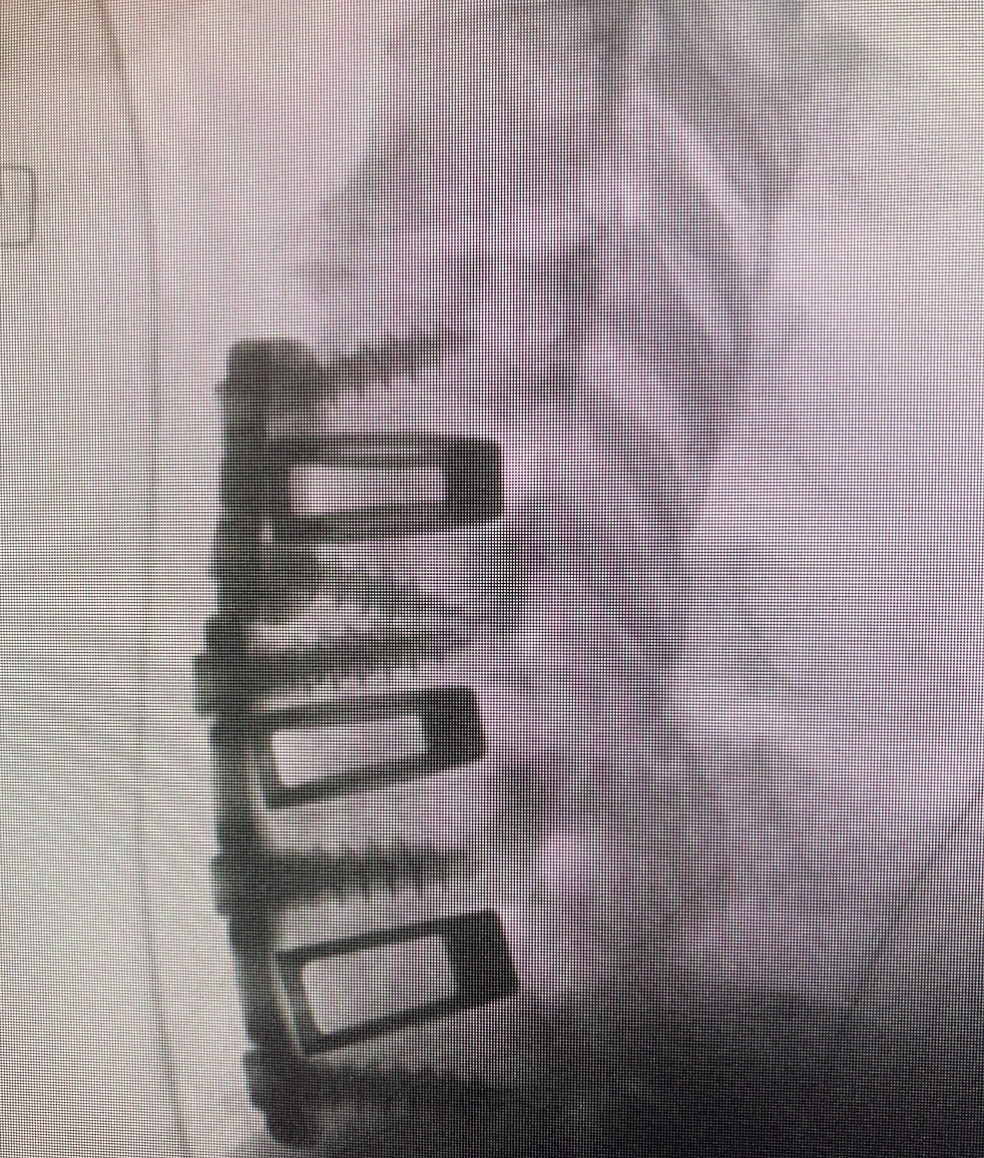
Lateral intraoperative C-spine fluoroscopy. This is an intraoperative lateral fluoroscopic image demonstrating the double-plate technique.

**Figure 8:**
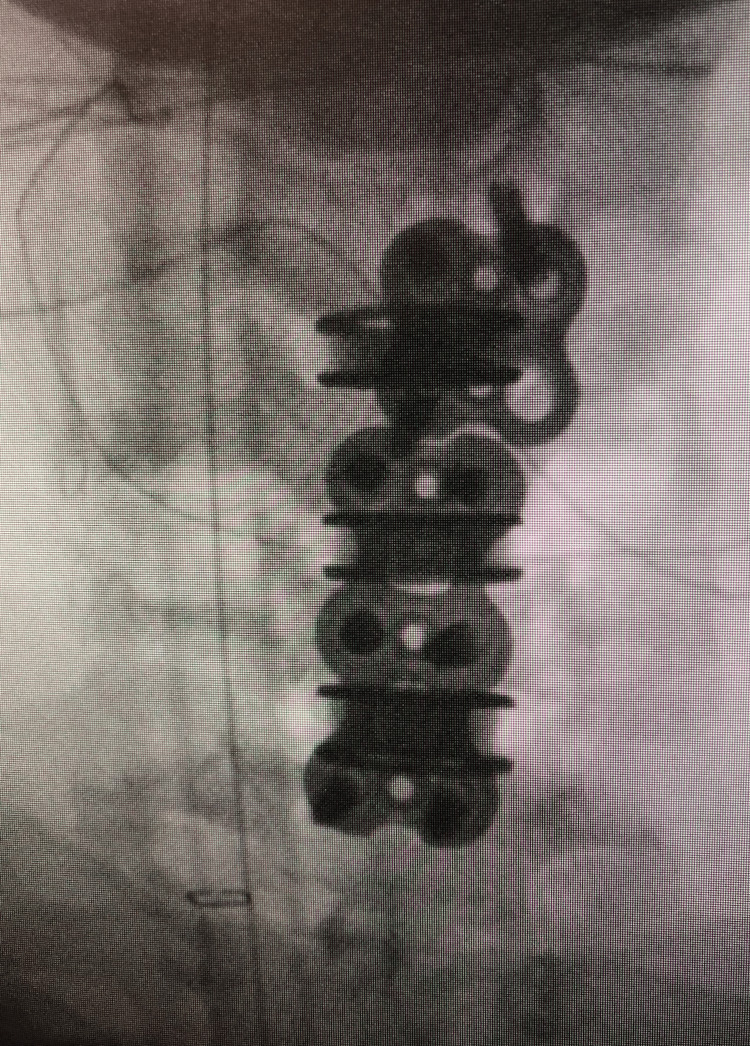
AP intraoperative fluoroscopy. This is an intraoperative AP fluoroscopic image demonstrating the double-plate technique. AP, anterior-posterior

The second patient was a 66-year-old male veteran with a BMI of 26.52 kg/m^2^ (1.70 m, 76.65 kg) with chief complaints of chronic neck pain (10/10) and left radicular arm pain (8/10). On physical examination, he had decreased sensation to light touch following the left C6-C7 dermatomal distribution. Magnetic resonance imaging (MRI) revealed multilevel cervical spondylosis from C3 to C7, with moderate-to-severe central and foraminal stenosis from C4 to C7. Figure 6 demonstrates his preoperative sagittal T2-weighted MRI. Electromyography (EMG) conduction study revealed fibrillation potential, which suggested active denervation of the C6 and C7 nerve roots. The patient had failed conservative treatment, including physical therapy, anti-inflammatory medications, and multiple epidural steroid injections. The patient would require a C4-C7 ACDF. The concern, however, was that the patient had severe spondylosis at the level of C3-C4. Even though he did not currently have significant stenosis at C3-C4 necessitating immediate surgical intervention, a future extension of fusion to C3-C4 may be anticipated. Therefore, a double-plate technique was used to aid future planning of a revision strategy, should it be needed. The patient underwent C4-C7 ACDF, with the top plate spanning C4-C5 and the bottom plate spanning C5-C7. Figures 7-8 demonstrate his intraoperative fluoroscopy imaging. The patient reported an improvement in VAS from 100 preoperatively to 40 postoperatively at one-year follow-up.

## Discussion

Although traditional usage of a single plate in multilevel ACDF is an effective treatment for several cervical spine pathologies, including cervical herniated discs, degenerative disc disease, and stenosis, the use of a double-plate technique offers several advantages [8-10]. Multiple finite-element studies support the usage of multiple plates with the potential to improve ACDF’s ability to restore natural lordosis, lower the risk of screw pull-out, and decrease plate strain [6,11]. Additionally, multiple-plate fixation may positively affect stiffness, maintain the range of motion (ROM) of adjacent segments, and mitigate construct failure and adjacent segment degeneration. In their biomechanical study of artificial cervical spine sawbones and cadaveric specimens, Rios and Eastlack found cervical spines stabilized with multiple individual segmental plates retained significantly more stiffness throughout fatigue testing when compared to spines stabilized with long cervical plates in axial rotation [7]. Additionally, they found no difference in flexion extension and lateral flexion between constructs.

Advantages

Increased Biomechanical Stability 

Using multiple plates instead of a single long plate may result in greater structural stability. Cervical spines with multiple plate constructs demonstrate superior stiffness during fatigue testing compared to spines stabilized with a long plate construct [6]. Multiple-plate constructs also require less pullout force ranging from 10 to 60 N, while a single long plate requires 35 to 82 N [11]. 

In their biomechanical study of cervical sawbones, Rios and Eastlack found that separated segmental anterior fixation for longer cervical fusions maintains its stiffness at the caudal aspect of the construct more effectively than a long single plate fixation [7]. They state this may lead to a reduction in mechanical and fusion complications, reduce the need for revisions or additional surgical approaches following surgery, and reduce risks of fracture, junctional kyphosis, fixation failure, and adjacent segment disease.

Decreased Retraction

Extensive traction is necessary to insert a long plate spanning multiple levels. This exposure can be reduced by using multiple plates, as they can be inserted without requiring the entire area to be exposed. Reducing the simultaneous exposure of a large area may decrease operative time and postoperative dysphagia [2,12,13]. Using multiple incisions following the skin crease may be more cosmetic compared to the traditional extensile approach. 

Revision Strategy 

During revision, it is easier to take out a shorter plate as opposed to a long plate. Using a shorter plate in the upper cervical spine and thoracolumbar junction may allow for easier revision if necessary. Also, placing the shorter plate adjacent to degenerated levels above or below the intended instrumented fusion site may decrease revision difficulty should the surgeon need to perform an extension of fusion at a future date. 

Disadvantages

Financial Considerations

The added financial cost incurred by a multiple-plate technique is the principal disadvantage compared to a single-plate construct. Using two plates instead of one plate may increase the cost of the implant between 50% and 80% for a three-level construct and between 30% and 50% for a four-level construct [14-16].

Axial Stress Load

The multiple-plate construct may induce a high axial stress load on the segment between the two constructs, as is especially demonstrated by case 2. This highlights the need for further investigation regarding the biomechanical considerations for the multiple-plate construct expanding on existing biomechanical studies [6,7,11].

## Conclusions

A multiple-plate technique may be effective in a long-level anterior cervical reconstruction. One needs to balance the potential advantages of this double-plate technique, increased biomechanical stability, decreased retraction, and ease of future revision, to the added financial costs compared to a single-plate technique. Both patients described in this report demonstrated improvements in VAS of 60 points, which is greater than the anticipated minimal clinically important difference. 
